# Development of an Innovative Intradermal siRNA Delivery System Using a Combination of a Functional Stearylated Cytoplasm-Responsive Peptide and a Tight Junction-Opening Peptide

**DOI:** 10.3390/molecules21101279

**Published:** 2016-09-24

**Authors:** Hisako Ibaraki, Takanori Kanazawa, Yuuki Takashima, Hiroaki Okada, Yasuo Seta

**Affiliations:** Laboratory of Pharmaceutics and Drug Delivery, Department of Pharmaceutical Science, School of Pharmacy, Tokyo University of Pharmacy and Life Sciences, 1432-1 Horinouchi, Hachioji, Tokyo 192-0392, Japan; ibaraki@toyaku.ac.jp (H.I.); takasima@toyaku.ac.jp (Y.T.); okada@toyaku.ac.jp (H.O.); setayas@toyaku.ac.jp (Y.S.)

**Keywords:** drug delivery, transdermal, siRNA, peptide, atopic dermatitis

## Abstract

As a new category of therapeutics for skin diseases including atopic dermatitis (AD), nucleic acids are gaining importance in the clinical setting. Intradermal administration is noninvasive and improves patients′ quality of life. However, intradermal small interfering RNA (siRNA) delivery is difficult because of two barriers encountered in the skin: intercellular lipids in the stratum corneum and tight junctions in the stratum granulosum. Tight junctions are the major barrier in AD; therefore, we focused on functional peptides to devise an intradermal siRNA delivery system for topical skin application. In this study, we examined intradermal siRNA permeability in the tape-stripped (20 times) back skin of mice or AD-like skin of auricles treated with 6-carboxyfluorescein-aminohexyl phosphoramidite (FAM)-labeled siRNA, the tight junction modulator AT1002, and the functional cytoplasm-responsive stearylated peptide STR-CH_2_R_4_H_2_C by using confocal laser microscopy. We found that strong fluorescence was observed deep and wide in the epidermis and dermis of back skin and AD-like ears after siRNA with STR-CH_2_R_4_H_2_C and AT1002 treatment. After 10 h from administration, brightness of FAM-siRNA was significantly higher for STR-CH_2_R_4_H_2_C + AT1002, compared to other groups. In addition, we confirmed the nontoxicity of STR-CH_2_R_4_H_2_C as a siRNA carrier using PAM212 cells. Thus, our results demonstrate the applicability of the combination of STR-CH_2_R_4_H_2_C and AT1002 for effective intradermal siRNA delivery.

## 1. Introduction

Intradermal drug administration is commonly used because of its easy application and removal of applied drugs. The skin consists of three layers: epidermis, dermis, and hypodermis. Macrophages, Langerhans cells, and mast cells related to the immune response are found in the epidermis and dermis. It is speculated that intradermal administration improves treatment by efficiently triggering small interfering RNA (siRNA) to these intradermal inflammatory cells. However, the epidermis contains strong barriers, including subcorneal intercellular lipid structures and tight junctions in the stratum granulosum. The intradermal delivery of hydrophilic macromolecules like siRNA is therefore difficult, necessitating the use of absorption enhancers.

Several recent studies have reported that not only the physical methods (such as microneedles, electroporation, or iontophoresis [1,2,3]) but also nanocarriers (such as liposome, polymer or carbon nanotubes, transfersome, and niosome) have the ability of penetrating siRNAs in the skin [4,5,6,7]. In addition, it is expected that siRNAs′ penetration efficiency can be improved by using cell-penetrating peptides [8,9]. Atopic dermatitis (AD) is a skin disease characterized by inflammation and itching, which is exacerbated by the drying that accompanies the breakdown of skin barriers. As no causative therapy is available, symptomatic treatment remains the only practical approach for managing AD [10,11].

In recent years, nucleic acids including siRNAs are receiving considerable attention as new AD therapeutics because of few side effects and potential for gene-level control of the disease. In addition, siRNAs have been shown to control cytokine production in allergic reactions and improve internal immune responses [12]. siRNAs do not require nuclear translocation or a highly efficient repression of gene expression. However, siRNAs are prone to degradation in vivo and have low permeability [13]. Therefore, to develop relevant pharmaceutical products, a carrier is necessary to improve siRNA stability, safety, and effective delivery to a target tissue or cell. In addition, the complex should be able to suppress the target gene efficiently. Cell-penetrating peptides (CPP) have been noted as effective siRNA carriers. Representative peptides used as gene carriers include peptides rich in basic amino acids (Tat peptides) and oligoarginines.

In a previous study, we reported a functional block peptide consisting of Cys-His-His-Arg-Arg-Arg-Arg-His-His-Cys (CH_2_R_4_H_2_C). Arginine has high cellular uptake, histidine can escape the endosome by the proton sponge effect, and cysteine forms stable complexion and can decondense in the cell [14]. A stearic acid (STR) modification at the N-terminus of this peptide (STR-CH_2_R_4_H_2_C) forms a nanocomplex with siRNA, stabilized by electrostatic interactions and disulfide cross linkages. The addition of the stearyl group to the peptide is expected to induce cellular uptake through high cellular affinity and stabilizing hydrophobic interactions. We have shown that STR-CH_2_R_4_H_2_C strikingly enhances in vitro and in vivo siRNA silencing efficiency [15].

In general, barrier functions of AD skin weaken or are disrupted. Changes in skin adipose composition due to the decrease in ceramide and sebaceous gland function and breaking of stratum corneum has been reported [13,16,17]. Therefore, the largest obstacles for siRNA delivery in AD patients are the tight junctions at the epidermis stratum granulosum. Tight junctions are cell junctions that connect neighboring cells and control the paracellular pathway of molecules. AT1002 is a hexamer synthetic peptide (H-FCIGRL-OH), which reversibly opens the tight junctions of the stratum granulosum and has biological activity similar to ΔG and zonula occludens toxin, increasing the paracellular transport of drugs across the epithelial barrier [18,19,20,21]. We have reported that the application of AT1002 can reversibly open tight junctions and increase intradermal siRNA delivery [22]. The deeper penetration of siRNA can expand the capability of treatment for skin diseases like AD, because the target cells of AD, like Langerhans cells, exists in the dermis [23].

In this study, we observed and analyzed the distribution of 6-carboxyfluorescein-aminohexyl phosphoramidite (FAM)-labeled siRNA (FAM-siRNA) in the back skin of tape-stripped normal mice and in the auricle skin of AD-like mice with FAM-siRNA using the stearylated cytoplasm-responsive peptide STR-CH_2_R_4_H_2_C and the tight junction opener peptide AT1002. Furthermore, we studied the toxicity of STR-CH_2_R_4_H_2_C as a siRNA carrier.

## 2. Results

### 2.1. Permeability of FAM-siRNA in Barrier-Disrupted Back Skin of Mice Using STR-CH_2_R_4_H_2_C and AT1002

**Figure 1:**
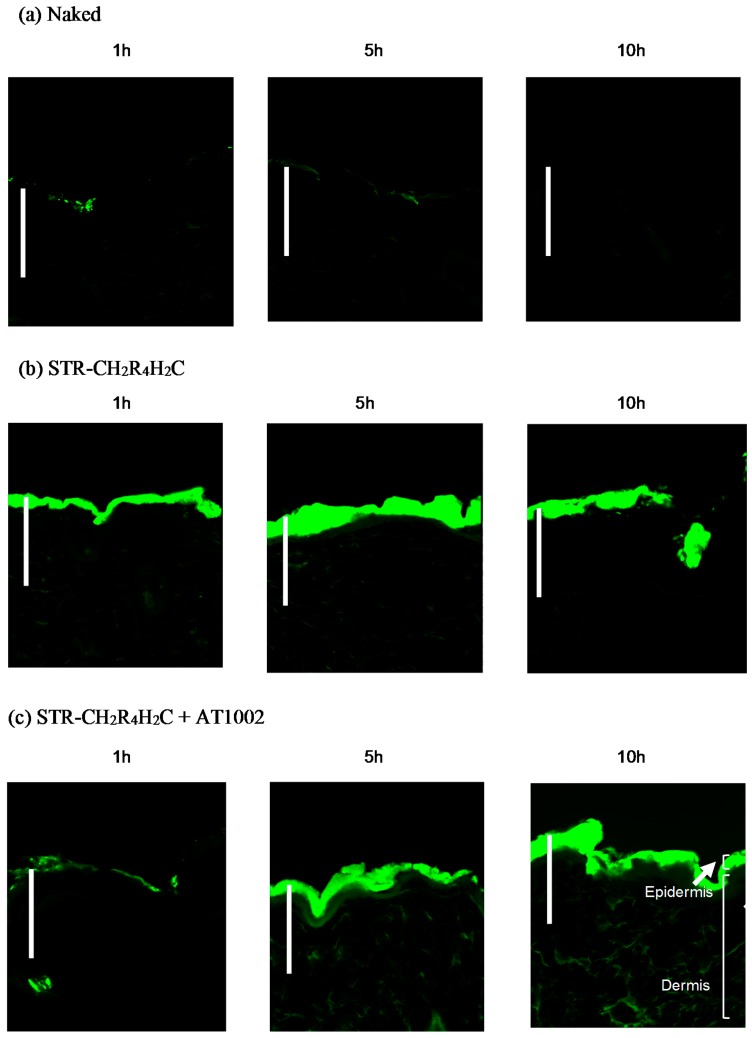
Permeability of 6-carboxyfluorescein-aminohexyl phosphoramidite (FAM)-labeled small interfering RNA (siRNA) using STR-CH_2_R_4_H_2_C + AT1002. (**a**) FAM-siRNA naked (5 μg); (**b**) FAM-siRNA (5 μg) with STR-CH_2_R_4_H_2_C; (**c**) FAM-siRNA (5 μg) with STR-CH_2_R_4_H_2_C + AT1002 (100 μg) were applied to the back skin of normal ICR mice for 1, 5, or 10 h. The skin sections (10 μm) were observed by confocal laser microscopy. Scale bar: 50 µm. Magnification: ×600.

We investigated the intradermal FAM-siRNA delivery using STR-CH_2_R_4_H_2_C and AT1002 in tape-stripped back skin of normal mice after treatment for 1, 5, or 10 h with naked FAM-siRNA, STR-CH_2_R_4_H_2_C, and STR-CH_2_R_4_H_2_C + AT1002 solutions (Figure 1). As shown in Figure 1a, no FAM fluorescence was observed in skin sections of naked FAM-siRNA-treated mice at any time point studied. In STR-CH_2_R_4_H_2_C with FAM-siRNA treatment, the FAM-siRNA fluorescence was observed in the epidermis and dermis, demonstrating that skin permeability could be improved using the stearic acid-modified STR-CH_2_R_4_H_2_C peptide (Figure 1b). In addition, in combined STR-CH_2_R_4_H_2_C and AT1002 treatment, the strongest fluorescence was observed in deeper sites of the dermis (approximately 50 µm from epidermis) compared to the STR-CH_2_R_4_H_2_C group (Figure 1c). After 1 h of treatment with siRNA/STR-CH_2_R_4_H_2_C + AT1002, the FAM-siRNA was mainly concentrated in the epidermis. After 5 h, fluorescence was observed in deeper sites of the stratum corneum and dermis. Among treatments studied, the STR-CH_2_R_4_H_2_C + AT1002 combination was the most effective for intradermal siRNA delivery.

**Figure 2:**
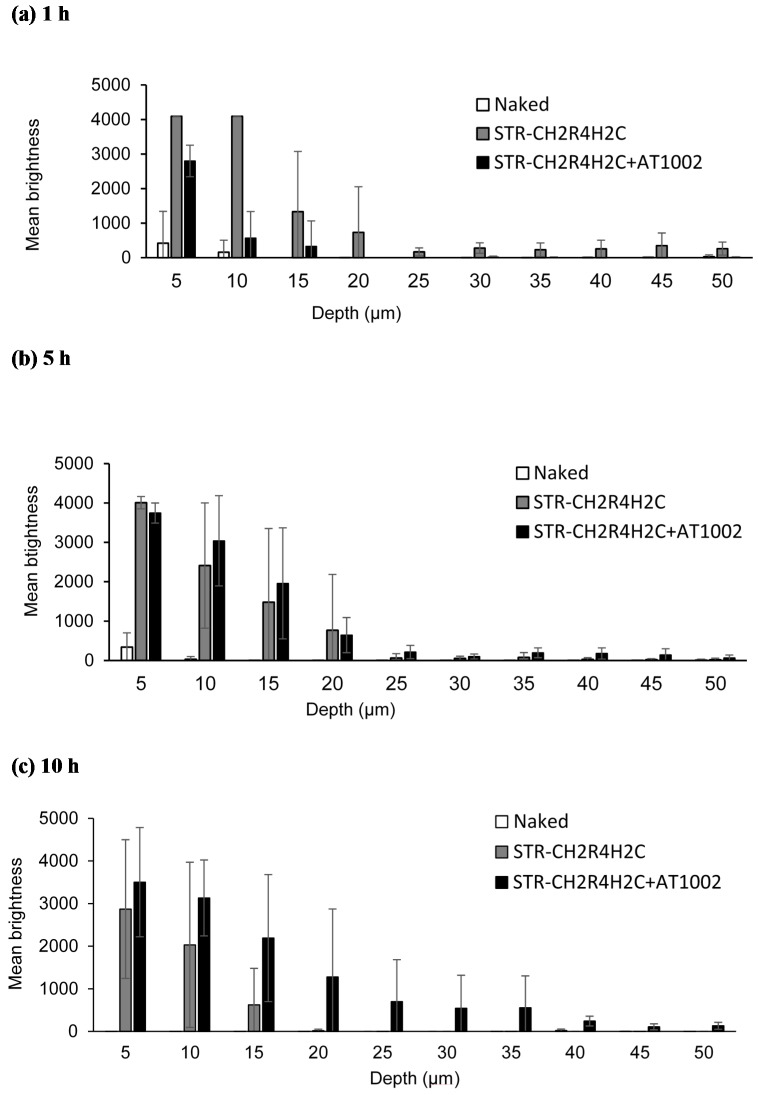
The mean brightness of FAM-labeled siRNA over time in tape-stripped mice. Naked FAM-siRNA (5 μg), FAM-siRNA (5 μg) with STR-CH_2_R_4_H_2_C (N/P ratio 10), and FAM-siRNA (5 μg) with STR-CH_2_R_4_H_2_C (nitrogen/phosphorous (N/P) ratio 10) + AT1002 (100 μg) were applied to the backs of normal ICR mice (**a**) 1 h; (**b**) 5 h; (**c**) 10 h after application. We measured the brightness of FAM-siRNA at depths 5–50 µm from the skin surface using confocal laser microscopy (Figure 1 images). The image area was chosen at random. Each bar represents the mean ± SD (*n* = 10).

Figure 2 shows the mean brightness of FAM-siRNA 1, 5, and 10 h after application at skin depths of 5–50 μm, quantifying the permeability of FAM-siRNA. After 1 h, strong fluorescence was seen at 5–10 μm from the skin surface with the STR-CH_2_R_4_H_2_C treatment (Figure 2a). After 5 h, FAM-siRNA with STR-CH_2_R_4_H_2_C and STR-CH_2_R_4_H_2_C + AT1002 treatments showed bright fluorescence at deeper sites (Figure 2b). In contrast, 10 h after application, bright siRNA fluorescence was observed in FAM-siRNA/STR-CH_2_R_4_H_2_C + AT1002-treated mice. At deeper sites (50 µm from the surface), the mean brightness of FAM-siRNA was higher for the STR-CH_2_R_4_H_2_C + AT1002 carrier compared to STR-CH_2_R_4_H_2_C (Figure 2c). These results indicate that STR-CH_2_R_4_H_2_C + AT1002 carrier delivered higher amounts of siRNA deeper into the skin.

### 2.2. Permeability of FAM-siRNA in AD-like Mice

**Figure 3:**
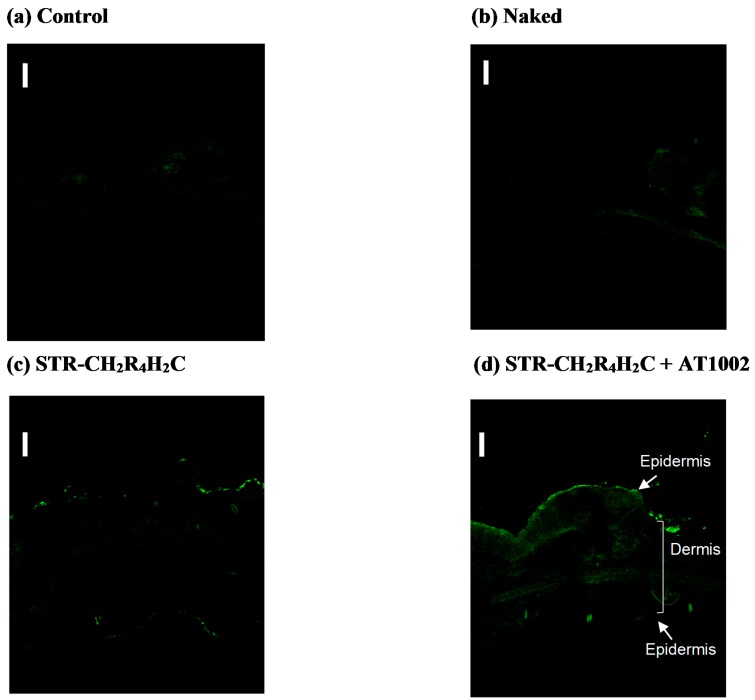
Permeability of FAM-labeled siRNA in NC/Nga atopic dermatitis (AD) model mice. Each FAM-siRNA solution listed below was applied to the skin of the left ears of AD model mice for 10 h. The skin sections (10 µm) were observed by confocal laser microscopy. Scale bar: 100 µm. Magnification: ×100. Applied FAM-siRNA solutions: (**a**) Untreated control; (**b**) Naked siRNA (5 µg); (**c**) FAM siRNA with STR-CH_2_R_4_H_2_C (65 µg); or (**d**) FAM-siRNA with STR-CH_2_R_4_H_2_C (65 µg) and AT1002 (100 µg).

Figure 3 shows the intradermal siRNA localization in AD-like mouse ears treated with AT1002 and STR-CH_2_R_4_H_2_C, 10 h after administration. In untreated control mouse ears (Figure 3a) and naked FAM-siRNA treated ears (Figure 3b), there was no fluorescence in the epidermis. In mouse ears treated with FAM-siRNA and STR-CH_2_R_4_H_2_C + AT1002 (Figure 3d), strong FAM-siRNA fluorescence was observed in the dermal tissue of the entire auricle. These results indicate that STR-CH_2_R_4_H_2_C + AT1002 complex enhanced siRNA delivery in AD mouse ears, where the stratum corneum barrier was broken.

**Figure 4:**
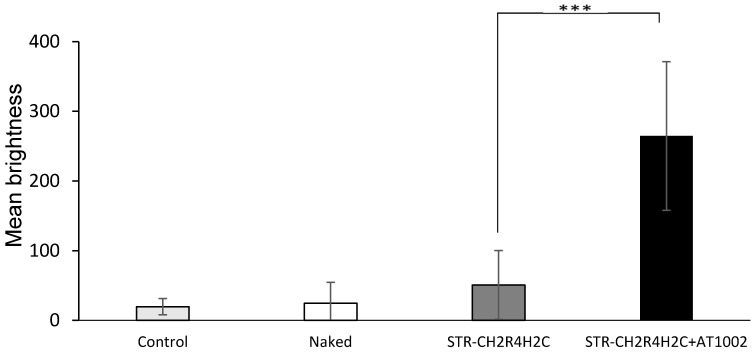
The mean brightness of FAM-labeled siRNA in NC/Nga AD model mice. Naked FAM-siRNA (5 μg), FAM-siRNA (5 μg) with STR-CH_2_R_4_H_2_C (N/P ratio 10), FAM-siRNA (5 μg) with STR-CH_2_R_4_H_2_C (N/P ratio 10) + AT1002 (100 μg) was applied to the left ears of NC/Nga AD model mice for 10 h. We measured the brightness of FAM-siRNA in the whole ear that received FAM-siRNA treatment using confocal laser microscopy Figure 3 images. *** *p* < 0.001 vs. other groups (*t*-test). Each bar represents the mean ± SD (*n* = 10).

Figure 4 shows the mean brightness of FAM-siRNA in AD-like mouse ears after 10 h of treatment, from the images shown in Figure 3. Similar to the results obtained with normal tape-stripped mouse skin, in AD-like mice, 10 h after administration of STR-CH_2_R_4_H_2_C + AT1002, FAM-siRNA could be observed in all layers of the skin. These findings suggest that the STR-CH_2_R_4_H_2_C + AT1002 complex is a promising carrier for intradermal delivery of siRNA.

### 2.3. In Vitro Cytotoxicity of STR-CH_2_R_4_H_2_C as siRNA Carrier

**Figure 5:**
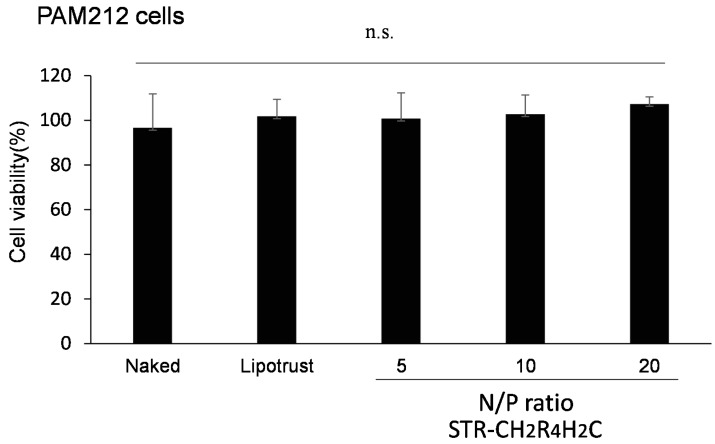
In vitro toxicity of STR-CH_2_R_4_H_2_C in PAM212 cells. Naked siControl, Lipotrust^®^, and siControl with STR-CH_2_R_4_H_2_C (N/P ratio 10–30) were transfected into PAM cells without serum. WST-8 assay was carried out 4 h after transfection. Each bar represents the mean ± SD (*n* = 6 wells). n.s. *p* > 0.05 versus nontreated control (*t-*test).

WST-8 assays showed that negative control siRNA siControl/STR-CH_2_R_4_H_2_C complexes at any nitrogen/phosphorous (N/P) ratio did not show any cytotoxicity in PAM212 cells (Figure 5), indicating that STR-CH_2_R_4_H_2_C is a nontoxic and safe siRNA carrier.

## 3. Discussion

In recent years, siRNA-based treatments for molecular-level control of diseases have been gaining popularity. Owing to sequence specificity, only a few doses can suppress disease-causing protein expression in allergic conditions. However, intradermal naked siRNA delivery is difficult owing to low permeability and stability of skin barriers such as the stratum corneum and tight junctions.

In recent studies, the noninvasive transdermal system has been attracting more attention, such as using the liposome, niosome, transfersome, and the cell-penetrating peptides. Arginine-rich peptides like Tat, because of their basic amine groups, have been reported to improve the intradermal delivery of proteins, peptides, and RNA interference (RNAi) agents [24,25,26,27]. Therefore, we propose that the arginine content of STR-CH_2_R_4_H_2_C improved the delivery of RNAi agents in this study, whereas the increased skin affinity was caused by the addition of the stearyl group. However, the underlying mechanisms of peptide-mediated delivery of RNAs across skin barriers are not fully understood, warranting further experiments. 

We previously reported that AT1002 opened tight junctions 10 h after treatment [27]; hence, this carrier may penetrate the skin from tight junction-opening regions, via paracellular and transcellular routes. AT1002 is a hexamer peptide and opens tight junctions reversibly by binding to zonulin and the PAR-2 receptor. AT1002 was suggested to promote the phosphorylation of the tight junction-related protein ZO-1, thereby reversibly opening tight junctions over time.

We aimed to develop an effective intradermal siRNA delivery system using a multifunctional stearylated peptide, STR-CH_2_R_4_H_2_C, and tight junction modulator peptide, AT10002. Because of the disrupted stratum corneum of AD patients, tight junctions in the stratum granulosum are the largest obstacle to siRNA delivery. The fluorescence of the FAM-siRNA/STR-CH_2_R_4_H_2_C + AT1002 complex was observed in the epidermis and dermis, unlike in the untreated and naked siRNA groups at 10 h after administration in tape-stripped normal mice (Figure 1 and Figure 2). In addition, STR-CH2 + AT1002 complex also enhanced siRNA delivery in actual AD-model mouse ears (Figure 3 and Figure 4). This result indicates that the combination of STR-CH_2_R_4_H_2_C and AT1002 enhances the delivery of siRNA into the dermis due to the penetrability of the functional peptide and opening of tight junctions caused by AT1002 (Figure 1, Figure 2, Figure 3 and Figure 4). Further studies are needed in order to indicate the mechanism of AT1002 or obtain more detail on the penetration of siRNA using this carrier. We would like to consider this in further research.

Figure 5 shows the cytotoxicity of STR-CH_2_R_4_H_2_C in vitro. STR-CH_2_R_4_H_2_C, at different N/P ratios, was confirmed to be a noncytotoxic and safe siRNA carrier, as indicated by WST-8 cell viability assay in PAM212 cells.

In conclusion, a combination of STR-CH_2_R_4_H_2_C and AT1002 provides an excellent siRNA carrier for topical administration. The use of STR-CH_2_R_4_H_2_C RNAi agents as topical therapeutics avoids systemic side effects and can enhance compliance and improve patient satisfaction. In addition, this useful and effective intradermal administration system might be applicable for management of other allergic conditions and skin diseases.

## 4. Materials and Methods

### 4.1. Peptides and siRNAs

The amino acid sequences of AT1002 and STR-CH_2_R_4_H_2_C peptides are shown in Table 1. The peptides were purchased from BEX Co. Ltd. (Tokyo, Japan).

The FAM-siRNA and negative control siRNA (siControl) were obtained from CosmoBio Co., Ltd. (Tokyo, Japan). The sequences of the sense and antisense oligonucleotides are shown in Table 2.

### 4.2. Animals

Six-week-old male ICR mice and NC/Nga mice were purchased from SLC (Hamamatsu, Japan). The mice were housed under standard condition (temperature: 23.5 ± 1 °C; humidity: 55% ± 5%) and a 12 h light/dark cycle. Food and water were supplied ad libitum. All experiments with animals were carried out in accordance with the protocol approved by the Animal Care and Ethics Committee of Tokyo University of Pharmacy and Life Sciences (the project identification code was P15-65 and the date of approval was 18 May in 2015).

### 4.3. Preparation of the siRNA Complex

First, we prepared each solution of siRNA (siRNA (5 µg)/UltraPure™ DNase/RNase-Free Distilled Water (12.5 µL) and STR-CH2R4H2C (65 µg)/UltraPure™ Water (12.5 µL)). Each solution (25 µL) at a nitrogen/phosphorous (N/P) ratio of 10 was mixed and incubated for 30 min at room temperature before use. These complexes were obtained by mixing equal quantities of siRNA solution and siRNA/STR-CH_2_R_4_H_2_C solution using the solvent UltraPure™ DNase/RNase-Free Distilled Water (Invitrogen Co., Waltham, MA, USA). The N/P ratio shows the charge ratio, and the cationic charge of zeta potential increases with N/P ratio.

### 4.4. In Vivo Study of FAM-siRNA Intradermal Permeation

Six-week-old male ICR mice were anesthetized by intraperitoneal administration of pentobarbital (20 mg/kg). The dorsal surface hairs were removed by an electric clipper and a cream-based hair remover (Kanebo, Tokyo, Japan). The backs of the mice were tape-stripped 20 times by surgical tape (3M Japan Limited, Tokyo, Japan), and 5 μg of FAM-siRNA samples (25 µL) (naked FAM-siRNA, siRNA/STR-CH_2_R_4_H_2_C, and siRNA/STR-CH_2_R_4_H_2_C + AT1002) was applied to the backs of the mice. Peptide quantities used were 100 and 65 µg for AT1002 and STR-CH_2_R_4_H_2_C, respectively. After 1, 5, and 10 h, the mice were sacrificed, and dermal tissues were washed with phosphate-buffered saline (PBS) and 1 cm^2^ samples were cut at the application site. The patch tester (Torii Pharmaceutical Co., Ltd., Tokyo, Japan) was applied to the treated area. The tissues were soaked in Tissue Mount (Siraimatsu, Osaka, Japan) at 4 °C in the dark, overnight. The tissues were preserved at −40 °C. In order to examine the skin permeability of siRNAs, frozen tissue sections were cut into 10 μm pieces by a Cryostat HM550 (Thermo Fisher Scientific Inc., Waltham, MA, USA). The sections were washed with PBS, incubated, and mounted with Fluorescence Mounting Medium (Dako Japan Inc., Tokyo, Japan). FAM-labeled siRNA in the skin was observed using confocal laser microscopy (FV1000D IX81, Olympus Corporation, Tokyo, Japan). Brightness was measured per fixed area at different depths (5–50 µm) from the surface of the skin.

### 4.5. Preparation of AD-Like Mice Model

Model AD skin was induced by repeated topical application of 2,4-dinitrofluorobenzene (DNFB; Wako Pure Chemical Ind. Ltd., Osaka, Japan) on NC/Nga mice for 15 days. NC/Nga mice were first sensitized with 0.15% DNFB solution (dissolved in ethanol/acetone 3:1) by topical application onto the left ear auricle (25 µL) and to the back skin (100 µL) removed after anesthetization with pentobarbital (20 mg/kg) on days 0 and day 4. After the first sensitization, 0.2% DNFB dissolved in acetone (25 µL) was applied to the left ear on days 7 and 10. The left ear was covered with olive oil after every administration.

### 4.6. Observation of FAM-siRNA in Auricle Skin of AD Model Mice

NC/Nga AD model mice were used in this study 15 days after the first sensitization. All mice were anesthetized by intraperitoneal administration of pentobarbital (20 mg/kg). FAM-labeled siRNA samples (25 µL of naked siRNA, siRNA/STR-CH_2_R_4_H_2_C, and siRNA/STR-CH_2_R_4_H_2_C + AT1002) were applied to each side of the auricles of the left ears. After 10 h, the mice were sacrificed, and the ear auricles were washed by PBS and resected. The tissues were soaked in Tissue Mount at 4 °C in the dark overnight and mounted with Tissue Mount in acetone and dry ice. The tissues were preserved at −40 °C. To examine the permeability of siRNAs in the ear lobes of AD model mice, 10-µm long frozen sections of the left ear lobe were prepared with a Cryostat HM550 (Thermo Fisher Scientific Inc.) and fixed in cold acetone. The slides were washed with PBS three times, washed with water, and mounted with a discoloration inhibitor. FAM-siRNA in the left ear auricle sections was observed using confocal laser microscopy.

### 4.7. Cell Preculture

PAM212, mouse keratinocytes were obtained from Dr. Hiroshi Matsuda (Tokyo University of Agriculture and Technology). The cells were precultured to 70%–80% confluence in Dulbecco′s Modified Eagle′s Medium (DMEM; GIBCO, Life Technologies, Thermo Fisher Scientific Inc.) containing 10% fetal bovine serum (FBS) and 1% penicillin/streptomycin (stock 10,000 U/mL and 10,000 µg/mL, respectively; Invitrogen Co.) before in vitro transfection studies at 37 °C in a humidified 5% CO_2_ atmosphere.

### 4.8. The Cytotoxicity of STR-CH_2_R_4_H_2_C

PAM212 (10^4^ cells/well) in 200 mL of DMEM containing 10% FBS were seeded onto 96-well plates and incubated. After 24 h, the cells were washed with PBS and transfected with naked-siControl, Lipotrust^®^ transfection agent (Hokkaido System Science Co., Ltd., Hokkaido, Japan), and siControl/STR-CH_2_R_4_H_2_C (N/P 5, 10, or 20) (0.2 µg of siRNA). After transfection for 4 h, CCK-8 (Dojindo laboratories, Kumamoto, Japan) solution was added to each well and the cells were incubated for 3 h. The absorbance of the cells in each well was measured using a microplate reader at 450 nm. The absorbance of control cells was set as the 100% viability standard, and the viability of all cells was expressed as a percentage relative to the absorbance of the control cells.

### 4.9. Statistical Analysis

The results of the experiments are represented as means ± standard errors. Statistical analysis of the data was performed using analysis of variance followed by t-test. Statistical significance was defined as highly significant (*** *p* < 0.001).

## Figures and Tables

**Table 1:** Peptide sequences.

Peptide	Sequence
AT1002	Phe-Cys-Ile-Gly-Arg-Leu
STR-CH_2_R_4_H_2_C	CH_3_(CH_2_)_16_CO-Cys-His-His-Arg-Arg-Arg-Arg-His-His-Cys

**Table 2:** Sequences of siRNAs.

Name		Sequence
FAM-labeled siRNA (FAM-siRNA)	Sense	5′-6-FAM AUC CGC GCG AUA GUA CGU AdTdT-3′
	Antisense	5′-UAC GUA CUA UCG CGC GGA UdTdT-3′
siControl	Sense	5′-AUC UGU GAG AUA GUA UGU AdTdT-3′
	Antisense	5′-UAC GUA CUA UCG CGC GGA UdTdT-3′

## References

[1] Tokumoto S., Higo N., Todo H., Sugibayashi K. (2016). Effect of Combination of Low-Frequency Sonophoresis or Electroporation with Iontophoresis on the Mannitol Flux or Electroosmosis through Excised Skin. Biol. Pharm. Bull..

[2] Kigasawa K., Kajimoto K., Hama S., Saito A., Kanamura K., Kogure K. (2010). Noninvasive delivery of siRNA into the epidermis by iontophoresis using an atopic dermatitis like model rat. Int. J. Pharm..

[3] Sivaraman A., Banga A.K. (2016). Novel in situ forming hydrogel microneedles for transdermal drug delivery. Drug Deliv. Transl. Res..

[4] Schlich M., Lai F., Murgia S., Valenti D., Fadda A.M., Sinico C. (2016). Needle-free jet injection of intact phospholipid vesicles across the skin: A feasibility study. Biomed. Microdevices.

[5] Kong M., Park H., Feng C., Hou L., Cheng X., Chen X. (2013). Construction of hyaluronic acid noisome as functional transdermal nanocarrier for tumor therapy. Carbohydr. Polym..

[6] Haigh O., Depelsenaire A.C., Meliga S.C., Yukiko S.R., McMillan N.A., Frazer I.H., Kendall M.A. (2014). CXCL1 gene silencing in skin using liposome-encapsulated siRNA delivered by microprojection array. J. Control. Release.

[7] Siu K.S., Chen D., Zheng X., Zhang X., Johnston N., Liu Y., Yuan K., Koropatnick J., Gillies E.R., Min W.P. (2014). Non-covalently functionalized single-walled carbon nanotube for topical siRNA delivery into melanoma. Biomaterials.

[8] Hsu T., Mitragotri S. (2011). Delivery of siRNA and other macromolecules into skin and cells using a peptide enhancer. Proc. Natl. Acad. Sci. USA.

[9] Morry J., Ngamcherdtrakul W., Gu S., Goodyear S.M., Castro D.J., Reda M.M., Sangvanich T., Yantasee W. (2015). Dermal delivery of HSP47 siRNA with NOX4-modulating mesoporous silica-based nanoparticles for treating fibrosis. Biomaterials.

[10] Leung D.Y. (2000). Atopic dermatitis: New insights and opportunities for therapeutic intervention. J. Allergy Clin. Immunol..

[11] Weidinger S., Novak N. (2016). Atopic dermatitis. Lancet.

[12] Kim D.H., Rossi J.J. (2009). Overview of gene silencing by RNA interference. Curr. Protoc. Nucleic Acid Chem..

[13] Bos J.D., Meinardi M.M. (2000). The 500 Dalton rule for the skin penetration of chemical compounds and drugs. Exp. Dermatol..

[14] Futaki S., Ohashi W., Suzuki T., Niwa M., Tanaka S., Ueda K., Harashima H., Sugiura Y. (2001). Stearylated arginine-rich peptides: A new class of transfection systems. Bioconjug. Chem..

[15] Tanaka K., Kanazawa T., Ogawa T., Takashima Y., Fukuda T., Okada H. (2010). Disulfide crosslinked stearoyl carrier peptides containing arginine and histidine enhance siRNA uptake and gene silencing. Int. J. Pharm..

[16] Jensen J.M., Proksch E. (2009). The skin’s barrier. G. Ital. Dermatol. Venereol..

[17] Mócsai G., Gáspár K., Dajnoki Z., Tóth B., Gyimesi E., Bíró T., Maródi L., Szegedi A. (2015). Investigation of skin barrier functions and allergic sensitization in patients with hyper-IgE syndrome. J. Clin. Immunol..

[18] Li M., Oliver E., Kitchens K.M., Vere J., Alkan S.S., Tamiz A.P. (2008). Structure-activity relationship studies of permeability modulating peptide AT-1002. Bioorg. Med. Chem. Lett..

[19] Song K.-H., Fasano A., Eddington N.D. (2008). Enhanced nasal absorption of hydrophilic markers after dosing with AT1002, a tight junction modulator. Eur. J. Pharm. Biopharm..

[20] Song K.-H., Fasano A., Eddington N.D. (2008). Effect of the six-mer synthetic peptide (AT1002) fragment of zonula occludens toxin on the intestinal absorption of cyclosporin A. Int. J. Pharm..

[21] Song K.-H., Kim S.B., Shim C.K., Chung S.J., Kim D.D., Rhee S.K., Choi G.J., Kim C.H., Kim K. (2015). Paracellular permeation-enhancing effect of AT1002 C-terminal amidation in nasal delivery. Drug Des. Dev. Ther..

[22] Uchida T., Kanazawa T., Kawai M., Takashima Y., Okada H. (2011). Therapeutic effects on atopic dermatitis by anti-RelA short interfering RNA combined with functional peptides Tat and AT1002. J. Pharmacol. Exp. Ther..

[23] Dubrac S., Schmuth M., Ebner S. (2010). Atopic dermatitis: the role of Langerhans cells in disease pathogenesis. Immunol. Cell Biol..

[24] Rothbard J.B., Garlington S., Lin Q., Kirschberg T., Kreider E., McGrane P.L., Wender P.A., Khavari P.A. (2000). Conjugation of arginine oligomers to cyclosporin A facilitates topical delivery and inhibition of inflammation. Nat. Med..

[25] Hou Y.W., Chan M.H., Hsu H.R., Liu B.R., Chen C.P., Chen H.H., Lee H.J. (2007). Transdermal delivery of proteins mediated by non-covalently associated arginine-rich intracellular delivery peptides. Exp. Dermatol..

[26] Robbins P.B., Oliver S.F., Sheu S.M., Goodnough J.B., Wender P., Khavari P.A. (2002). Peptide delivery to tissues via reversibly linked protein transduction sequences. BioTechniques.

[27] Uchida T., Kanazawa T., Takashima Y., Okada H. (2011). Development of an efficient transdermal delivery system of small interfering RNA using functional peptides, Tat and AT-1002. Chem. Pharm. Bull..

